# Performance of the BG1Luc ER TA Method in a qHTS Format

**DOI:** 10.14573/altex.1505121

**Published:** 2015-06-28

**Authors:** Patricia Ceger, David Allen, Ruili Huang, Menghang Xia, Warren Casey

**Affiliations:** 1 integrated Laboratory Systems, Inc., Research Triangle Park, North Carolina, USA; 2 National Chemical Genomics Center, National Center for Advancing Translational Sciences, National Institutes of Health, Rockville, Maryland, USA; 3 National Toxicology Program Interagency Center for the Evaluation of Alternative Toxicological Methods, National Institute of Environmental Health Sciences, Research Triangle Park, North Carolina, USA

**Keywords:** BG1Luc ER TA, quantitative high-throughput screening, Tox21, EDSP

## Abstract

In 2012, the BG1Luc4E2 estrogen receptor (ER) transactivation (TA) method (BG1Luc ER TA) was accepted by U.S. regulatory agencies and the Organisation for Economic Co-operation and Development to detect substances with ER agonist activity. The method is now part of the Tier 1 testing battery in the Environmental Protection Agency’s Endocrine Disruptor Screening Program. The BG1Luc ER TA method uses the BG1 ovarian cell line that endogenously expresses full-length ER (α and β) and is stably transfected with a plasmid containing four estrogen responsive elements upstream of a luciferase reporter gene. To allow increased throughput and testing efficiency, the BG1Luc ER TA (“BG1 manual”) method was adapted for quantitative high-throughput screening (BG1 qHTS) in the U.S. Tox21 testing program. The BG1 qHTS test method was used to test approximately 10,000 chemicals three times each, and concentration-response data (n = 15) were analyzed to evaluate test method performance. The balanced accuracy of the BG1 qHTS test method (97%, i.e., 32/33) was determined by comparing results to ER TA performance standards for the BG1 manual method. Concordance between the BG1 manual and qHTS methods was 92% (57/62) when calculated for a larger set of non-reference chemicals tested in both methods. These data demonstrate that the performance of the BG1 qHTS is similar to the currently accepted BG1 manual method, thereby establishing the utility of the BG1 qHTS method for identifying ER active environmental chemicals.

## 1 Introduction

The U.S. federal Tox21 consortium was formed to address the research agendas described in the National Research Council report ‘Toxicity Testing in the 21^st^ Century: A Vision and a Strategy” (NRC, 2007). Tox21 is a collaboration of the National Toxicology Program (NTP), the National Center for Advancing Translational Sciences (NCATS) of the National Institutes of Health (NIH), the National Center for Computational Toxicology (NCCT) of the Environmental Protection Agency (EPA), and the Food and Drug Administration. The Tox21 partner agencies have worked together to develop, validate and translate innovative *in vitro* high-throughput screening (HTS) methods to characterize the impact of chemicals on key steps in toxicity pathways. Methods to be included in the Tox21 collaboration must be adapted to the NCATS robotic platform for quantitative high-throughput screening (qHTS) (Michael et al., 2008; Attene-Ramos et al., 2013) and are used to screen a 10,000-chemical library, available in a titration-based format (Attene-Ramos et al., 2013).

Toxicity associated with the endocrine system is of particular interest for Tox21, since exposure to “endocrine active chemicals” (EACs) that mimic hormones can result in developmental or reproductive problems (ICCVAM, 2011; Rinckel et al., 2014). Given the paucity of data available on endocrine activity for the vast majority of chemicals in commerce, there is a particular need for a fast and efficient mechanism to collect information that could be used in prioritizing the chemicals of greatest concern for further assessment. EACs may affect growth and development through a variety of mechanisms associated with a multitude of hormone pathways. Three hormone pathways, estrogen, androgen and thyroid, are the focus of the EPA’s Endocrine Disruptor Screening Program (EDSP). Of these three signaling pathways, those involved with interaction with the estrogen receptor are particularly well-characterized and a number of test methods that target them have been developed.

The BG1Luc4E2 estrogen receptor (ER) transactivation (TA) test method (BG1Luc ER TA) was evaluated in an international interlaboratory validation study coordinated by the NTP Interagency Coordinating Committee for the Evaluation of Alternative Toxicological Methods (NICEATM), in partnership with the European Union Reference Laboratory for Alternatives to Animal Testing and the Japanese Center for the Evaluation of Alternative Methods. The BG1Luc ER TA method uses the BG1Luc4E2 human ovarian adenocarcinoma cell line that is stably transfected with an estrogen-responsive luciferase reporter gene to measure TA activity via ER-mediated pathways (Rogers and Denison, 2000, 2002). Based on the validation study results, performance standards were developed for the BG1Luc ER TA method to evaluate the comparability of subsequent proposed test methods that are functionally and mechanistically similar (ICCVAM, 2011). These performance standards included a list of 34 reference substances for assessing the sensitivity and specificity of proposed test methods. The BG1Luc ER TA method and associated performance standards were reviewed and accepted by member agencies of the Interagency Coordinating Committee on the Validation of Alternative Methods (ICCVAM), including the EPA. The BG1Luc ER TA method is now accepted as one component of Tier 1 testing in the EPA’s EDSP. As part of Tier 1 screening, the method is used as an independent part of a weight-of-evidence approach to prioritize potentially endocrine-active substances for further testing and, as such, does not directly reduce, refine or replace animal use. However, the data generated during Tier 1 testing is used to determine the need for further *in vivo* Tier 2 testing (EPA, 2011), which may reduce the number of substances tested in Tier 2 and thereby reduce animal use.

The validated BG1Luc ER TA method (which we will subsequently refer to as “BG1 manual”) was successfully adapted to automated formats (Bittner et al., 2015; Stoner et al., 2014), nominated for inclusion in Tox21 and adapted to the NCATS qHTS format (BG1 qHTS). NCATS generated BG1 qHTS data for the Tox21 library of 10,000 test substances, 76 of which were also included in the BG1 manual validation study and are therefore the focus of this evaluation. This report describes an evaluation of this data to assess the degree to which classifications of test chemicals by the BG1 qHTS method matched (1) the classifications of chemicals in the ICCVAM performance standards (accuracy) and (2) classifications of the BG1 manual method for the 76 chemicals tested using both methods (concordance). This evaluation demonstrates the potential utility of the qHTS version of the validated BG1Luc ER TA test method and a basis for considering BG1 qHTS data for EPA Tier 1 EDSP testing.

## 2 Methods and materials

Detailed descriptions of the BG1 manual methods, including information regarding the availability of the cells, and complete assay protocols have been published (ICCVAM, 2011) and are available online^1^.

Detailed protocols for the BG1 qHTS method are going to be made available through the Tox21 consortium website^2^.

### Elements in common to both test methods

Both the manual and the qHTS methods use an ER-responsive luciferase reporter gene (luc) in the BG1Luc4E2 human ovarian adenocarcinoma cell line to detect substances with *in vitro* ER activity. ER-mediated transcription of the luc gene produces luciferase, which catalyzes the production of light from luciferin. Luminescence is measured and expressed in relative light units (RLU). For both the BG1 manual and qHTS agonist methods, the reference standard is 17β-estradiol. Seventy-six chemicals were tested in both the manual and qHTS methods. The qHTS results for the complete 10K library are discussed in Huang et al. (2014).

Key elements of the BG1 manual and qHTS methods and differences between the two methods are summarized in Table 1.

### BG1Luc ER TA manual test method

The BG1 manual test method and associated validation data are detailed in an ICCVAM test method evaluation report (ICCVAM, 2011). The BG1 manual method is performed in 96-well plates. Each 96-well plate contains an 11-point concentration-response curve for the reference standard and two test substances, as well as vehicle and positive controls. Each substance is analyzed in triplicate wells. Each plate is considered an independent experiment.

RLU values obtained from experiments are adjusted by subtracting background luminescence from control, reference and test substance wells. To define the upper limit for test substance concentrations, scores for cell viability are assigned using visual observation of numbers (density) and shapes (morphology) of cells. Control, reference and test substance RLU values are then adjusted relative to the highest reference standard RLU value, which is set to 10,000. After adjustment, values are transferred to GraphPad Prism® for data analysis (e.g., determination of half-maximal effective concentration (EC_50_) values) and graphing.

The validation study for the BG1 manual method (ICCVAM, 2011) included 78 substances tested in three different laboratories. Each substance was tested in three replicate wells in a 96-well plate in at least one experiment.

### Tox21 10K library of chemicals

The Tox21 10K library (Attene-Ramos et al., 2013) includes 11,776 substances (8188 possessing unique Chemical Abstracts Service Registry Numbers®). The substances in the library were nominated in approximately equal proportions by the EPA, NTP and the NIH Chemical Genomics Center (NCGC), and include substances from the NCGC Pharmaceutical Collection (Huang et al., 2011). The substances were prepared as stock solutions and then serially diluted in dimethylsulfoxide (DMSO) in 1536-well microplates to yield 15 concentrations ranging from 1.1 nM to 92 *μ*M (Attene-Ramos et al., 2013). As previously noted, the focus of this analysis was on only those chemicals for which both qHTS and manual data were available.

### BG1Luc ER TA qHTS test method

Guidance criteria for Tox21 methods are listed on the NCATS website included in NTP (2010). Briefly, methods submitted to the Tox21 program are optimized and miniaturized into a 1536-well plate format. Methods are initially validated using the LOPAC1280 library of 1280 pharmacologically active substances from Sigma-Aldrich run in triplicate (NTP, 2010). Method acceptance criteria include a Z’ factor (Zhang et al., 1999) greater than 0.5, a coefficient of variation less than 10%, and a signal to background ratio greater than 3 (NTP, 2010). Methods that meet these acceptance criteria are used to test the Tox21 10K library (Huang et al., 2014). However, exceptions have been made, e g., for the BG1 method, which has a signal to background ratio of 2.5, and another ER TA method, the HEK293 ER-bla antagonist assay, which has a Z’ factor of 0.4 (Huang et al., 2014).

NCATS used the BG1 qHTS method to test the complete Tox21 10K library as follows. A pin transfer station was used to transfer 23 nl of substance from a 1536-well source plate to a 1536-well method plate, with each plate holding up to 1408 test substances (located in columns 5-48). Method-specific controls (located in columns 1-4) were obtained from an additional 1536-well compound plate and transferred simultaneously with the test substances to the method plate (Michael et al., 2008; NTP, 2010). Each substance was run in a single well and single concentration per plate.

Once data were obtained for each method, they were normalized relative to a method-specific positive control (100%) and vehicle-only wells (0%). Normalized data were then corrected by applying a pattern-correction algorithm using data from vehicle-only plates that were tested at the beginning and end of each plate stack (Inglese et al., 2006; Shukla et al., 2010; Xia et al., 2008).

### Data analysis: BG1 manual method

In order to match the processing method used for the BG1 qHTS data, the BG1 manual data from the validation study were converted from RLU to percent response of the reference standard.

After conversion to percent of reference standard response, BG1 manual data were transformed to a log_10_ scale and graphed as concentration-response curves using GraphPad Prism® version 6.00 for Windows (GraphPad Software, Inc., San Diego, CA, USA). The graph for each substance was evaluated visually and classified as positive, negative or inconclusive as detailed in the BG1 manual test method evaluation report (ICCVAM, 2011). Specifically, substances that generated responses greater than three times the standard deviation of the vehicle control mean (3X SD) and produced a sigmoidal dose-response curve were classified positive. Responses less than 3X SD were classified negative. Substances for which a definitive positive or negative classification could not be determined because of poor quality data were considered inconclusive and not used in the performance evaluation. EC_50_ values were calculated for positive substances using the sigmoidal dose-response equation in GraphPad Prism®.

### Data analysis: BG1 qHTS method

BG1 qHTS data from the Tox21 program for comparison with the BG1 manual data were obtained from the EPA’s ToxCast program. ToxCast analyses of Tox21 data are freely available from the NCCT Computational Toxicology Research website, http://epa.gov/ncct/toxcast/data.html. The specific data analyses downloaded from NCCT were ToxCast_Tox21_Level5&6_20141022. Detailed descriptions of the analyses performed are included in the download package.

Analysis of compound concentration-response data was performed as previously described (EPA, 2012, 2014). In short, raw plate reads for each titration point were first normalized to the median plate raw values (serving as the negative control value) and the maximal (for agonist) bimodal response of the control wells. If the difference between the negative and positive control wells was less than three standard deviations (3x SD) across all plate-level raw values, then the median plus or minus 3x SD across all plate-level raw values was used and served as the positive control value. The percent activity was calculated as

$$(({\text{V}}_{\text{compound}}-{\text{V}}_{\text{negative}})/({\text{V}}_{\text{positive}}-{\text{V}}_{\text{negative}}))\times 100$$

where V_compound_ denotes compound well raw value, V_negative_ denotes median plate raw value, and V_positive_ denotes maximal (agonist) bimodal response peak among the control wells. Baseline correction and outlier detection were subsequently performed on the normalized percent activity values using a modification of a robust outlier detection methodology (Motulsky and Christopoulos, 2004). All surviving normalized percent activity values for each compound were fitted to three models: a constant model (no activity), a Hill model, and a “Gain-Loss” model, which is a combination of two Hill models, one increasing in signal, and the other decreasing at higher concentration. The Gain-Loss model helps account for observed loss of activity at high concentrations that is most likely due to cytotoxicity for certain substances. A response cutoff for each method was established using ten times the median absolute deviation of the first five tested concentrations across all tested substances. The top of the Hill model curve as well as the average measured response at a tested concentration had to surpass the response cutoff to be considered a potential “active.” Additional concentration-based filters then were applied to filter out confounded initial “active” classifications (EPA, 2014).

The fitting procedures used to evaluate BG1 qHTS data placed chemicals into “active” or “inactive” bins (i.e., there was no “inconclusive” bin). In calling actives, there was a balance between allowing false positives and false negatives. The procedure attempted to minimize both, but was qualitatively weighted towards allowing more false positives. Where multiple samples of the same chemical were run, the run that produced an active concentration of 50% (AC_50_) value (or the most potent AC_50_ where more than one was available) was used.

### Comparison of BG1 manual and BG1 qHTS results

**Fig. 1:**
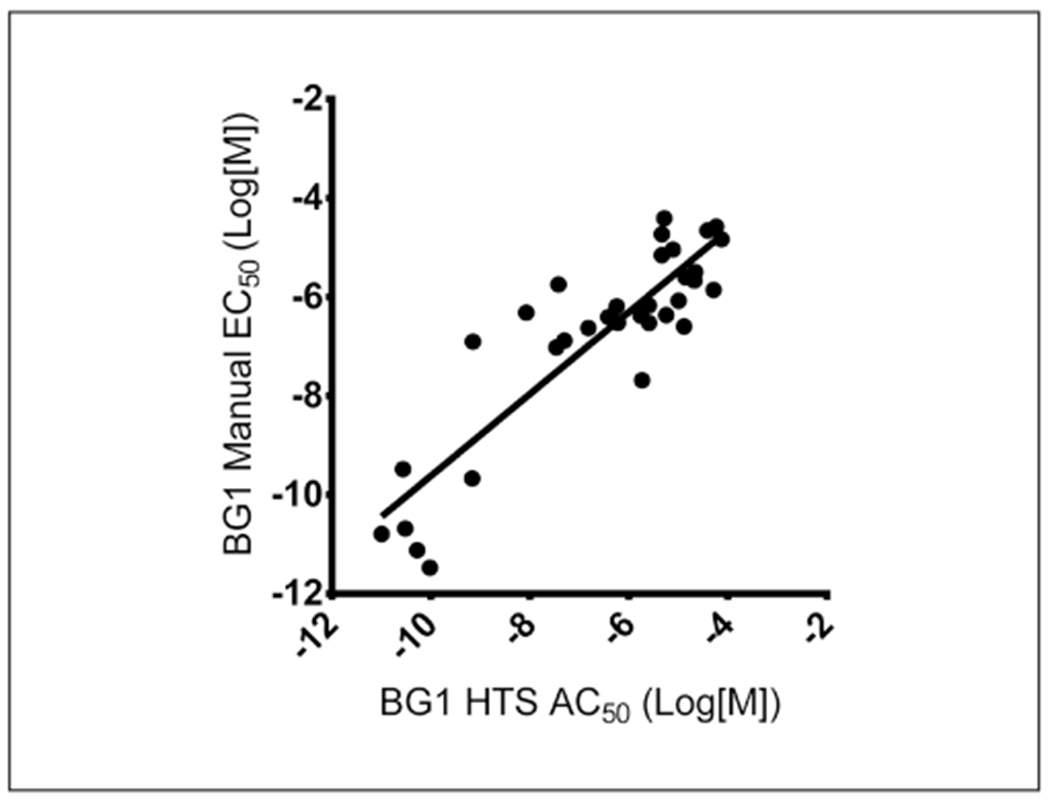
Linear regression analysis of BG1 manual and qHTS EC_50_/AC_50_ values A linear regression analysis was conducted of EC_50_/AC_50_ values for 33 substances that tested positive in the BG1 manual and HTS methods. A list of the chemicals used to create Figure 1 is included in Table 2. The slope of the linear regression is 0.48 with r^2^ of 0.69.

Linear regression analyses were used to compare the EC_50_ (manual) and AC_50_ (qHTS) values for all substances that tested positive in the manual and qHTS protocols (Fig. 1). A list of the chemicals used to create Figure 1 is included in Table 2.

The minimum list of 34 reference substances for assessing the accuracy of the proposed test method provided in the ICCVAM performance standards for the BG1 manual method (ICCVAM, 2011) is provided in Table 3. Accuracy of the BG1 qHTS method was calculated based on the degree to which results obtained using this method agreed with the ICCVAM performance standards classifications for the same substances.

Concordance between the BG1 manual and qHTS methods was determined comparing the results from all of the substances (64/76) that produced a definitive classification in the BG1 manual method to the classifications obtained from the qHTS data (Tab. 4) and evaluating the degree to which classifications of test chemicals were identical between the two methods (see supplementary file at http://dx.doi.org/10.14573/altex.1505121s).

A quantitative analysis was conducted for the BG1 manual and qHTS methods on a per-chemical basis and on an overall method basis (i.e., all EC_50_/AC_50_ values). For the per-chemical analysis, a paired t-test was conducted to determine whether the median EC_50_/AC_50_ values for the manual and qHTS methods were different (p < 0.05). For the overall analysis, a two-tailed Mann-Whitney U test was done to determine whether the calculated EC_50_/AC_50_ values differed significantly (p < 0.05). Because of the large dataset available specifically for the 17β-estradiol reference standard, we compared the EC_50_/AC_50_ values for each 17β-estradiol replicate. We also conducted a qualitative comparison of repeatability by evaluating results of replicate experiments based on curve shape and overall result (i.e., positive or negative).

## 3 Results

### 3.1 Data quality

BG1 manual method performance and data quality are detailed in the BG1 manual test method evaluation report (ICCVAM, 2011). qHTS method data quality, as evaluated by NCATS, was high as indicated by a coefficient of variation (< 10.5%), reproducibility (outcome matches across triplicate runs, ≥ 87%), and Z’ factor (≥ 0.5) (Huang et al., 2014). The 17β-estradiol positive control titrations embedded in every plate replicated well across the entire screen with standard deviations of AC_50_ varying by less than 3-fold.

### 3.2 BG1 qHTS test method accuracy

**Fig. 2:**
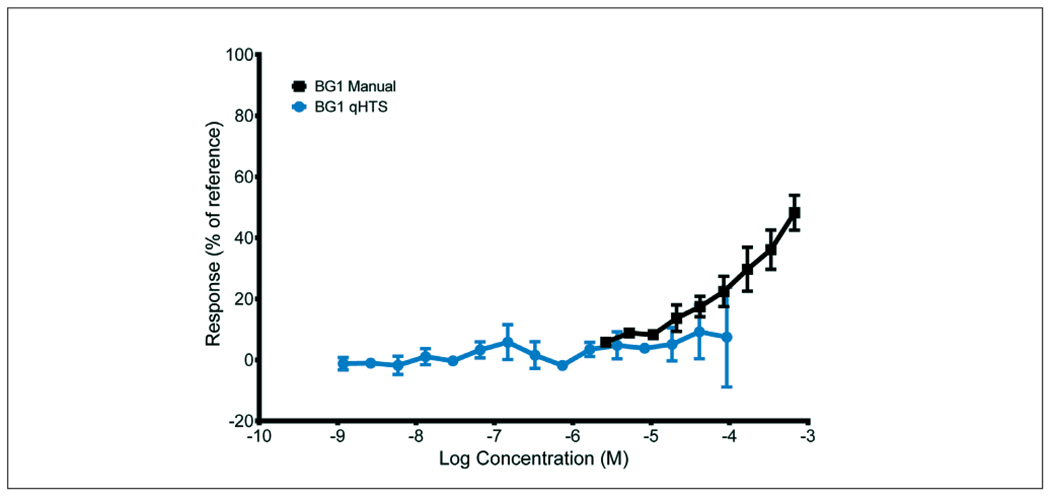
Discordant response for dicofol in BG1 manual and qHTS methods Concentration-response curves for dicofol tested with the agonist protocols for the BG1 manual and qHTS methods. The data plotted for BG1 manual represent results from a single laboratory, and each point is the mean of three within-experiment replicates (+/− standard deviation). The qHTS data represents the mean of three curves (one each in each experiment, +/− standard deviation).

BG1 qHTS classifications for 34 performance standards reference substances (27 positive and 7 negative), which were used for calculating test method accuracy, are shown in Table 3. Accuracy for the BG1 qHTS method was 97% (33/34). Dicofol, a positive reference substance, was misclassified as negative in the qHTS method. However, the positive result in the BG1 manual occurs at concentrations above the highest concentration tested in BG1 qHTS (92 *μ*M; Fig. 2).

### 3.3 Concordance

Of the 78 substances tested in the BG1 manual validation study, 76 (morin and 12-O-tetradecanoylphorbal-13-acetate were not included) were also tested in the BG1 qHTS method. Concordance was evaluated using a subset of these 76 substances. Test substances that were considered inconclusive in the BG1 manual method were omitted from analysis, leaving a total of 64 substances (see supplementary file at http://dx.doi.org/10.14573/altex.1505121s). Concordance between classifications produced by the BG1 manual and qHTS methods for these 64 substances was 92% (59/64) (Tab. 4). All five of the discordant substances (2-sec-butylphenol, dicofol, di-n-butyl phthalate, nilutamide and phenolphthalin) were positive in the BG1 manual method, but negative in the BG1 qHTS. The discordance for three of these substances (2-sec-butylphenol, dicofol, and phenolphthalin) could be explained by the fact that they were not tested at concentrations as high as those tested in the BG1 manual method.

### 3.4 Repeatability

The ToxCast_Tox21_Level5&6_20141022 download includes a quality statistics summary, which evaluates the overall repeatability between chemical replicates. This information is calculated as the percentage of times all BG1 qHTS classifications for a chemical were either negative or positive (e.g., 0 out of 3 or 3 out of 3) over the total number of chemicals with replicates. For the BG1 agonist method, the overall repeatability between chemical replicates was 0.79, indicating that there is some variability between chemical replicates.

We examined the BG1 qHTS classifications of each of the 64 substances that were evaluated for concordance. The overall repeatability between chemical replicates for this smaller dataset was 93% (60/64). Decreased repeatability across runs for four substances – di-*n*-butyl-phthalate, fenarimol, progesterone, and propylthiouracil – can be attributed primarily to different lots of chemical tested among the replicate tests.

### 3.5 Quantitative EC_50_/AC_50_ comparison

We also evaluated the quantitative differences in EC_50_/AC_50_ values for the 33 positive substances in both the BG1 manual and qHTS methods (Fig. 1). The slope of the linear regression presented in Figure 1 is 0.48 with an r^2^ of 0.69, indicating that, while qualitative classifications were identical for these substances, there were some quantitative differences on a per-chemical basis. However, although there were up to 100-fold differences in EC_50_/AC_50_ values for several substances, when compared on an individual chemical basis using a paired t-test there were no differences in EC_50_ values (p > 0.05). Likewise, when the population of EC_50_/AC_50^S^_ derived in the manual and qHTS methods were compared (i.e., median EC_50_/AC_50_ from all 33 chemicals) using a two-tailed Mann-Whitney U test, there was no quantitative difference (p = 0.35).

A Spearman correlation analysis was also performed to evaluate the rank order of substances between the two methods. The Spearman *r* value was 0.73 (p < 0.0001), indicating that the two methods correlate well.

However, when all 17β-estradiol reference standard EC_50_/AC_50_ values were compared, there were significant differences between those obtained using the BG1 manual and qHTS methods (p < 0.0001). Although the mean EC_50_ values for BG1 manual using values reported by all three laboratories in all four phases (n = 218, mean log EC_50_ = −11.08M) and qHTS (n = 801, mean log AC_50_ = −10.68 M) were within an order of magnitude, the standard deviations were small (0.58 and 0.18 for BG1 manual and qHTS, respectively), particularly given the size of the population, and therefore the difference is highly significant. However, these data also indicate that both methods are highly repeatable when testing the reference standard.

## 4 Discussion

The manual BG1 method, validated by NICEATM and recommended by ICCVAM, has been accepted by the EPA and OECD for regulatory use and included in the EDSP Tier 1 screening battery. One of the goals of the Tox21 program is to develop, validate and translate test methods that characterize toxicity pathways while reducing cost and animal use (Shukla et al., 2010; Huang et al., 2011). Tox21 is using qHTS methods to reach this goal. To that end, the manual BG1 method was adapted to the Tox21 qHTS format, thereby providing an opportunity to compare qHTS results to a validated manual method.

ICCVAM performance standards for the BG1Luc ER TA are intended for the evaluation of test methods that are functionally and mechanistically similar to the validated method and include a list of 34 reference chemicals with classifications for ER agonist activity (ICCVAM, 2011). The accuracy of the BG1 qHTS test method was evaluated by comparing the outcomes of tests of 34 reference substances with those published in the ICCVAM performance standards (ICCVAM, 2011). The accuracy of the BG1 qHTS method was 97% (33/34) (Tab. 3). Dicofol, a cyclic halogenated hydrocarbon pesticide, produced the only discordant result (false negative) when testing the performance standards substances. However, dicofol was tested in the BG1 manual method at concentrations above the highest concentration tested in the BG1 qHTS. As indicated in Figure 2, there is increased variability at the upper end of the concentration response curve for the BG1 qHTS method. This variability could very well be masking an upper trend that would mimic the shape of the BG1 manual curve. Considered along with the difference in concentration ranges tested, this information suggests that dicofol could test positive at higher concentrations. Regardless, the relevance of a positive result at such high concentrations in an *in vitro* system could be questioned as a false positive (ICCVAM, 2011) and thus it is noteworthy that the manual and qHTS results were in 100% agreement at ≤ 100*μ*M.

When considering definitive results for all substances tested in both the BG1 manual and qHTS methods, concordance between the two methods was 92% (59/64). The five discordant substances were all positive in the manual method, but negative in the qHTS. The discordant results do not appear to be due to variance between replicates, as of the four substances with decreased qHTS repeatability, only di-n-butyl phthalate was discordant between the manual and qHTS methods.

As noted above for test method accuracy, the discordance for three of these substances (2-sec-butylphenol, dicofol, and phenolphthalin) could be explained by the fact that they were not tested at higher concentrations to match the BG1 manual dose range. Furthermore, for each of these substances, results in the BG1 qHTS method display an increased variability at the highest concentrations that may mask an upward trend that would suggest they could test positive at higher concentrations. Di-n-butyl phthalate is classified as negative in BG1 qHTS, although there is an upward trend at concentrations equal to and greater than 10 *μ*M, suggesting that it could be considered a borderline response. Nilutamide is clearly negative in the qHTS method and clearly positive in the manual method, but it should be noted that there are no ER TA, ER binding or *in vivo* uterotrophic reference data that provide support for a definitive classification for this substance (Ceger et al., 2015, 2014; ICCVAM, 2011). The relevance of this discordant result is unclear.

Evaluation of the quantitative differences in EC_50_/AC_50_ values for the 33 positive substances in both the BG1 manual and qHTS methods indicated that while qualitative classifications were identical for these substances, there are up to 100-fold differences in EC_50_/AC_50_ values for several substances (Fig. 1 and Tab. 2). However, when the population of EC_50_/AC_50^S^_ derived in the manual and qHTS methods were compared, there was no significant difference, suggesting that the overall sensitivity of the BG1 method is similar whether in manual or qHTS format.

These data demonstrate that the performance of the BG1 qHTS method is similar to that of the BG1 manual method, thereby demonstrating the utility of qHTS for identifying potentially ER-active chemicals.

## Supplementary Material

7B3D6652031D06806ACA3AD751AE117B

## Figures and Tables

**Tab. 1:** Summary of differences between the BG1Luc ER TA manual and qHTS methods

	BG1 manual	BG1 qHTS
**Plate format**	96-well	1536-well
**Number of substances tested per plate**	2	1408
**Concentrations tested per plate**	11	1/plate X 15 plates
**Stock solutions and serial dilutions**	Stock solutions and serial dilutions were prepared for each experiment.	Stock solutions were provided by participating agencies. Serial dilutions were prepared for each experiment.
**Concentration-response curve**	Generated for each substance on a single plate	Generated over 15 plates
**Testing concentrations**	Determined by range finder, followed by focused testing (~3 log range) up to the limit of solubility or 1 mM	Fixed concentrations typically ranging from 1.1 nM to 92 μM
**Within-experiment replicates**	Each test substance tested in triplicate in each experiment	Each test substance tested once in each experiment
**Between-experiment replicates**	Each experiment performed at least twice	Each experiment performed in triplicate
**Plating density**	40,000 cells per well	4,000 cells per well
**Well volume**	200 μl per well	5 μl per well
**Well washing**	Wash steps	No wash steps
**Days to conduct experiment**	Up to 14	5 to screen the entire 10 K library
**Evaluation of cytotoxicity**	Determined by visual inspection	Not evaluated

**Tab. 2:** EC_50_ and AC_50_ values for the 33 positive substances in both the BG1 manual and qHTS methods

Test substance	CASRN	BG1 manual median EC_50_ value (log M)	BG1 qHTS median AC_50_ value (log M)
**17-ɑ estradiol**	57-91-0	−9.48	−10.56
**17-ɑ ethinyl estradiol**	57-63-6	−11.12	−10.28
**17-β estradiol**	50-28-2	−11.47	−10.02
**17β-trenbolone**	10161-33-8	−7.02	−7.47
**19-nortestosterone**	434-22-0	−5.74	−7.42
**4-cumylphenol**	599-64-4	−6.52	−6.22
**4-hydroxyandrostenedione**	566-48-3	−4.41	−5.29
**4-tert-octylphenol**	140-66-9	−7.68	−5.74
**Apigenin**	520-36-5	−5.85	−4.29
**Bisphenol A**	80-05-7	−6.40	−6.42
**Bisphenol B**	77-40-7	−6.63	−6.82
**Butylbenzyl phthalate**	85-68-7	−5.66	−4.68
**Chrysin**	480-40-0	−5.49	−4.66
**Coumestrol**	479-13-0	−6.88	−7.30
**Daidzein**	486-66-8	−6.17	−5.60
**Diethylstilbestrol**	56-53-1	−10.68	−10.52
**Estrone**	53-16-7	−9.67	−9.17
**Ethyl paraben**	120-47-8	−4.58	−4.24
**Fenarimol**	60168-88-9	−5.04	−5.11
**Flavone**	525-82-6	−5.15	−5.34
**Fluoranthene**	206-44-0	−4.83	−4.13
**Fluoxymestrone**	76-43-7	−4.65	−4.42
**Genistein**	446-72-0	−6.52	−5.59
**Kaempferol**	520-18-3	−6.59	−4.89
**Kepone**	143-50-0	−6.36	−5.25
**meso-hexestrol**	84-16-2	−10.79	−10.99
**Methyl testosterone**	58-18-4	−6.19	−6.25
**Norethynodrel**	68-23-5	−6.90	−9.15
***o,p’*-DDT**	789-02-6	−6.37	−5.76
***p.p*’-methoxychlor**	72-43-5	−6.07	−5.00
***p*-n-nonylphenol**	104-40-5	−5.60	−4.85
**Progesterone**	57-83-0	−4.73	−5.33
**Testosterone**	58-22-0	−6.31	−8.07

Table contains median EC_50_ and AC_50_ values for the 33 substances that were positive in both the BG1 manual and qHTS methods. Comparison of chemicals on an individual bases using a paired t-test indicated that there were no significant differences in EC_50_/AC_50_ values. Evaluation of the chemicals on a population basis using a two-tailed Mann-Whitney U test indicated no quantitative difference. CASRN, Chemical Abstracts Service Registry Number.

**Tab. 3:** BG1 qHTS classifications of performance standards substances

Test substance	CASRN	Performance standards classification	BG1 qHTS classification
**17-ɑ estradiol**	57-91-0	POS	POS
**17-ɑ ethinyl estradiol**	57-63-6	POS	POS
**17-β estradiol**	50-28-2	POS	POS
**19-nortestosterone**	434-22-0	POS	POS
**4-cumylphenol**	599-64-4	POS	POS
**4-*tert*-octylphenol**	140-66-9	POS	POS
**Apigenin**	520-36-5	POS	POS
**Bisphenol A**	80-05-7	POS	POS
**Bisphenol B**	77-40-7	POS	POS
**Butylbenzyl phthalate**	85-68-7	POS	POS
**Chrysin**	480-40-0	POS	POS
**Coumestrol**	479-13-0	POS	POS
**Daidzein**	486-66-8	POS	POS
**Dicofol**	115-32-2	POS	NEG
**Diethylstilbestrol**	56-53-1	POS	POS
**Estrone**	53-16-7	POS	POS
**Ethyl paraben**	120-47-8	POS	POS
**Fenarimol**	60168-88-9	POS	POS
**Genistein**	446-72-0	POS	POS
**Kaempferol**	520-18-3	POS	POS
**Kepone**	143-50-0	POS	POS
***meso*-hexestrol**	84-16-2	POS	POS
**Methyl testosterone**	58-18-4	POS	POS
**Norethynodrel**	68-23-5	POS	POS
***o,p’*-DDT**	789-02-6	POS	POS
***p,p’*-methoxychlor**	72-43-5	POS	POS
***p*-n-nonylphenol**	104-40-5	POS	POS
**Atrazine**	1912-24-9	NEG	NEG
**Bicalutamide**	90357-06-5	NEG	NEG
**Corticosterone**	50-22-6	NEG	NEG
**Hydroxyflutamide**	52806-53-8	NEG	NEG
**Linuron**	330-55-2	NEG	NEG
**Phenobarbital**	50-06-6	NEG	NEG
**Spironolactone**	52-01-7	NEG	NEG

Table contains positive/negative classifications for the list of 34 reference substances for assessing the sensitivity and specificity of proposed published test methods. CASRN: Chemical Abstracts Service Registry Number; NEG: negative; POS: positive

**Tab. 4:** Concordance of the agonist protocols for the BG1 manual and qHTS methods

	**BG1 qHTS classification**			
**BG1 manual classification**		**Positive**	**Negative**	**Total**
	**Positive**	33	5	38
	**Negative**	0	26	26
	**Total**	33	31	64

Concordance was evaluated for 64 substances (38 positive, 26 negative) tested using both the BG1 manual and qHTS methods, omitting substances that yielded inconclusive results in the BG1 manual method. Overall concordance between the two methods for the 64 substances was 92% (59/64).

## Footnotes

1
http://1.usa.gov/1IxwRyX

2
http://tox21.org/home
