# Supplementary material for: Performance of the BG1Luc ER TA Method in a qHTS Format
Source: ALTEX. Author manuscript; Available in PMC 2022 Apr 17. (PMC9013469; doi:10.14573/altex.1505121)
Supplement: 7B3D6652031D06806ACA3AD751AE117B [file NIHMS1788111-supplement-7B3D6652031D06806ACA3AD751AE117B.pdf]

Ceger et al.:

Performance of the BG1Luc ER TA Method in a qHTS Format

Supplementary Data

Tab. S1: BG1 manual and qHTS classifications for 64 chemicals with a definitive classification in the BG1 manual method

Test substance	CASRN	BG1 Manual Classification	BG1 qHTS Classification
2,4,5-trichlorophenoxy-acetic acid	93-76-5	NEG	NEG
4-hydroxytamoxifen	68047-06-3	NEG	NEG
Actinomycin D	50-76-0	NEG	NEG
Ammonium perchlorate	7790-98-9	NEG	NEG
Apomorphine	58-00-4	NEG	NEG
Atrazine	1912-24-9	NEG	NEG
Bicalutamide	90357-06-5	NEG	NEG
Corticosterone	50-22-6	NEG	NEG
Cyproterone acetate	427-51-0	NEG	NEG
Dibenzo(*a*,*h*) anthracene	53-70-3	NEG	NEG
Finasteride	98319-26-7	NEG	NEG
Haloperidol	52-86-8	NEG	NEG
Hydroxyflutamide	52806-53-8	NEG	NEG
Ketoconazole	65277-42-1	NEG	NEG
L-thyroxine	51-48-9	NEG	NEG
Linuron	330-55-2	NEG	NEG
Medroxy-progesterone acetate	71-58-9	NEG	NEG
Mifepristone	84371-65-3	NEG	NEG
Phenobarbital	50-06-6	NEG	NEG
Pimozide	2062-78-4	NEG	NEG

This is an Open Access article distributed under the terms of the Creative Commons Attribution 4.0 International license (http://creativecommons.org/licenses/by/4.0/), which permits unrestricted use, distribution and reproduction in any medium, provided the original work is appropriately cited.

http://dx.doi.org/10.14573/altex.1505121s

Test substance	CASRN	BG1 Manual Classification	BG1 qHTS Classification
Propylthiouracil	51-52-5	NEG	NEG
Raloxifene HCl	82640-04-8	NEG	NEG
Reserpine	50-55-5	NEG	NEG
Sodium azide	26628-22-8	NEG	NEG
Spironolactone	52-01-7	NEG	NEG
Vinclozolin	50471-44-8	NEG	NEG
2-sec-butylphenol	89-72-5	POS	NEG
Dicofol	115-32-2	POS	NEG
Nilutamide	63612-50-0	POS	NEG
Phenolphthalin	81-90-3	POS	NEG
Progesterone	57-83-0	POS	POS
Di-*n*-butyl phthalate	84-74-2	POS	NEG
17-α estradiol	57-91-0	POS	POS
17-α ethinyl estradiol	57-63-6	POS	POS
17-β estradiol	50-28-2	POS	POS
17β-trenbolone	10161-33-8	POS	POS
19-nortestosterone	434-22-0	POS	POS
4-cumylphenol	599-64-4	POS	POS
4-hydroxyandrostenedione	566-48-3	POS	POS
4-tert-octylphenol	140-66-9	POS	POS
Apigenin	520-36-5	POS	POS
Bisphenol A	80-05-7	POS	POS
Bisphenol B	77-40-7	POS	POS
Butylbenzyl phthalate	85-68-7	POS	POS
Chrysin	480-40-0	POS	POS
Coumestrol	479-13-0	POS	POS
Daidzein	486-66-8	POS	POS
Diethylstilbestrol	56-53-1	POS	POS
Estrone	53-16-7	POS	POS
Ethyl paraben	120-47-8	POS	POS

Test substance	CASRN	BG1 Manual Classification	BG1 qHTS Classification
Fenarimol	60168-88-9	POS	POS
Flavone	525-82-6	POS	POS
Fluoranthene	206-44-0	POS	POS
Fluoxymestrone	76-43-7	POS	POS
Genistein	446-72-0	POS	POS
Kaempferol	520-18-3	POS	POS
Kepone	143-50-0	POS	POS
meso-hexestrol	84-16-2	POS	POS
Methyl testosterone	58-18-4	POS	POS
Norethynodrel	68-23-5	POS	POS
o,p'-DDT	789-02-6	POS	POS
p,p'-methoxychlor	72-43-5	POS	POS
p-n-nonylphenol	104-40-5	POS	POS
Testosterone	58-22-0	POS	POS

CASRN: Chemical Abstracts Service Registry Number; IC: Inconclusive;
NEG: negative; POS: positive.
Table contains positive/negative classifications for the 76 substances tested in common between the BG1 manual and BG1 qHTS assays.
